# First experimental evidence for a bis-ethene chromium(I) complex forming from an activated ethene oligomerization catalyst

**DOI:** 10.1126/sciadv.abd7057

**Published:** 2020-12-18

**Authors:** S. Chabbra, D. M. Smith, N. L. Bell, A. J. B. Watson, M. Bühl, D. J. Cole-Hamilton, B. E. Bode

**Affiliations:** 1 EaStCHEM School of Chemistry, University of St Andrews, St Andrews, Fife KY16 9ST, Scotland, UK.; 2 Centre of Magnetic Resonance, University of St Andrews, St Andrews, Fife KY16 9ST, Scotland, UK.; 3 Drochaid Research Services, St Andrews, Fife KY16 9ST, Scotland, UK.

## Abstract

A bis-ethene chromium(I) species, which is the postulated key intermediate in the widely accepted metallacyclic mechanism for ethene oligomerization, is experimentally observed. This catalytic transformation is an important commercial route to linear α-olefins (primarily, 1-hexene and 1-octene), which act as comonomers for the production of polyethene. Here, electron paramagnetic resonance studies of a catalytic system based on [Cr(CO)_4_(PNP)][Al(OC(CF_3_)_3_)_4_] [PNP = Ph_2_PN(^i^Pr)PPh_2_] activated with Et_6_Al_2_ provide the first unequivocal evidence for a chromium(I) bis-ethene complex. The concentration of this species is enhanced under ethene and isotope labeling studies that confirm its composition as containing [Cr(C_2_H_4_)_2_(CO)_2_(PNP)]^+^. These observations open a new route to mechanistic studies of selective ethene oligomerization.

## Teaser

Isotope labeling and electron paramagnetic resonance spectroscopy reveal a long-postulated bis-ethene chromium(I) motif.

## INTRODUCTION

We report the first bis-ethene chromium(I) complex. These complexes have been postulated for over 40 years as the key intermediate in the metallacyclic mechanism of commercialized selective ethene oligomerization reactions (*1*), but none has ever been identified experimentally.

**Fig. 1:**
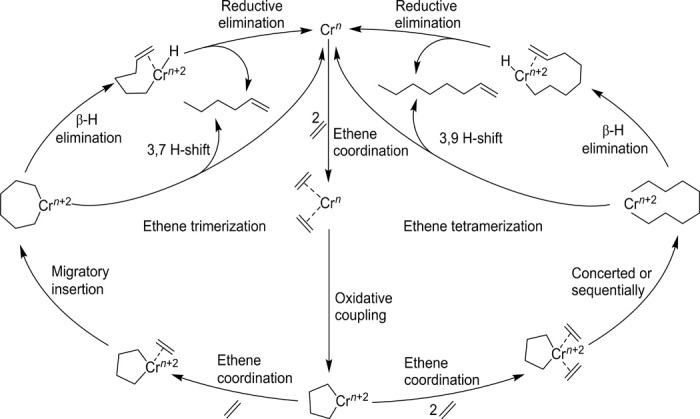
Proposed metallacyclic and extended metallacyclic mechanisms. The formation of 1-hexene and 1-octene are shown here for ethene trimerization and tetramerization, respectively.

Linear α-olefins (LAOs) are valuable chemical building blocks for the production of surfactants, lubricants, and detergents, but they are most importantly used as comonomers for high-density and linear low-density polyethene (*2*, *3*). Polyethene is one of the most widely used plastics in consumer products such as food packaging, coatings, bags, toys, power cable coatings, gas pipes, etc., and continues to replace other materials (*4*, *5*). A total of 3.5 million tons of LAOs was produced in 2012, rising with a growth rate of about 3.3% per year (2012–2018) projected to reach a $11.5 billion market by 2025 (*6*). Even numbered LAOs are commonly produced by ethene oligomerization (Ziegler, modified Ziegler, Shell higher olefins, and Fischer-Tropsch processes) (*2*, *7*, *8*) with statistical product distributions (i.e., Schulz-Flory and Poisson distributions) including the formation of higher polymers hampering continuous flow operation (*9*, *10*). However, increasing demand for specific alkenes (1-hexene and 1-octene) makes selective production highly desirable. Chromium catalysts give rise to selective 1-hexene formation, and different systems have been commercialized on the basis of their high activity and selectivity (*1*, *11*, *12*). A metallacyclic trimerization mechanism (Fig. 1) has initially been postulated from kinetic data (*1*). Deuterium isotope labeling studies (*13*) support this mechanism, the expanded mechanism for formation of 1-octene, and abundant by-products (*14*). The postulated mechanisms (Fig. 1) involve the following: (i) η^2^ coordination of two ethene molecules, (ii) oxidative coupling to give a metallacyclopentane, (iii) coordination and migratory insertion of further ethene irreversibly forming larger metallacycles (*13*), and (iv) either an H-shift (*14*) or a β-hydride elimination and reductive elimination to afford the LAO and regenerate the catalyst. Trimerization and tetramerization stem from a single catalyst precursor (*15*) with the selectivity of tetramerization catalysts guided by accessibility for further ethene and the relative stabilities of seven- and nine-membered metallacycles (*14*, *16*). Computational (*17*) and experimental (*18*, *19*) data back a Cr^I/III^ (*20*–*22*) redox couple. Chromium(I) ethene complexes and five- and seven-membered chromium(III) metallacycles have been observed (*23*), but the key bis-ethene complex has remained elusive until this work.

The most selective catalyst system for ethene tetramerization is generated in situ by activating a mixture of Cr^III^ precursor and diphosphinoamine [Ph_2_PN(^i^Pr)PPh_2_ = PNP] ligand with an excess of modified methylaluminoxane (*12*). The formation of a cationic chromium(I)/PNP species (*20*) stabilized by a weakly coordinating anion (MAO-Me)¯ is a crucial step. Cationic chromium(I)/PNP complexes with weakly coordinating anions show efficient tetramerization catalysis (*20*), making them attractive for mechanistic investigations. Electron paramagnetic resonance (EPR) spectroscopy is exclusively and exquisitely sensitive to paramagnetic centers and ideally suited for monitoring a Cr^I/III^ redox couple. EPR studies revealed the formation of deactivating sandwich complexes of chromium(I) in toluene solvent strongly correlating with diminished catalyst performance (*24*, *25*).

A chromium(I) precursor was selected, as it enables highly resolved EPR spectra while it has been shown to lead to the same catalytical activity as the common and economically more viable chromium(III) precursors (*20*). In this work, chromium(I)-precatalyst **1** (**1** = [Cr(CO)_4_(PNP)][Al(OC(CF_3_)_3_)_4_]) was activated in the absence and presence of ethene gas and the Cr speciation was monitored by EPR. Selective isotope labeling of carbonyl ligands and ethene gas gives strong evidence for the composition of a novel intermediate.

## RESULTS

**Fig. 2:**
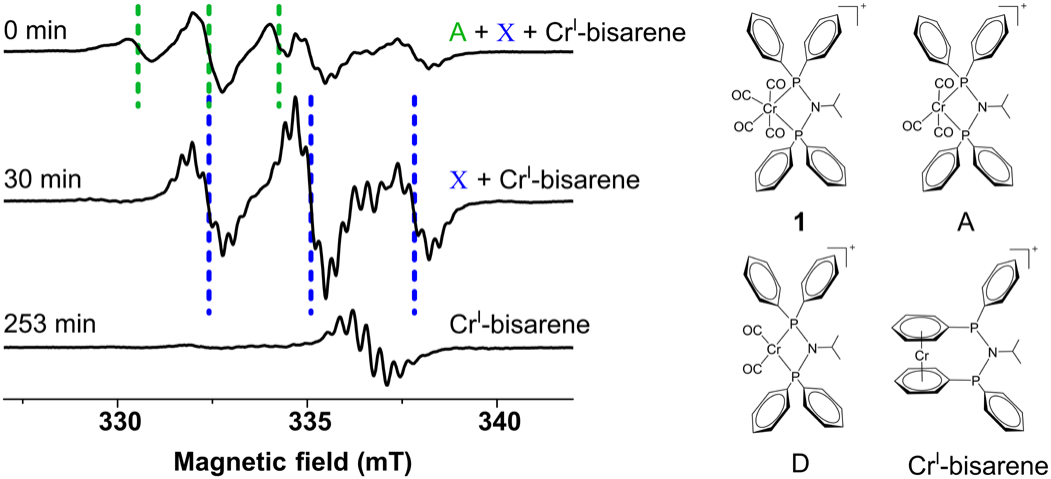
EPR data showing formation of three different species over time. **1** dissolved in a 1:1 solvent mixture of dichloromethane (DCM) and methylcyclohexane (MCH) by volume was activated in situ at 273 K by adding 5 equivalents of Et_6_Al_2_. The continuous-wave (CW) EPR spectra were recorded every minute for 16 hours at 295 K. Individual time slices are shown with field positions marked by dashed lines showing the following: (i) A (0 min, green), (ii) X (30 min, blue), and (iii) Cr^I^-bisarene (253 min). The structures of **1** and previously reported species (*26*) A and D and Cr^I^-bisarene are presented.

**Fig. 3:**
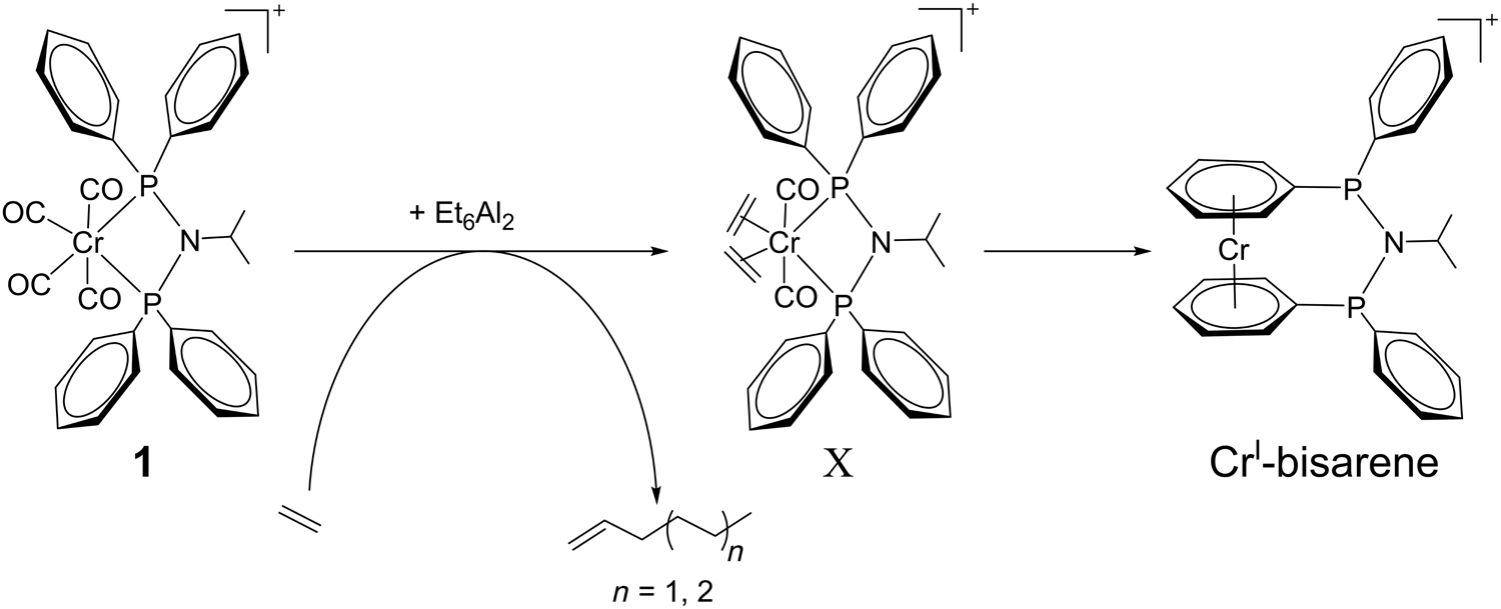
Proposed mechanism showing intermediates formed upon activation. The transformation of Cr^I^-precatalyst **1** upon activation with 5 equivalents of Et_6_Al_2_ in a solvent mixture of 1:1 ratio of DCM and MCH by volume.

**1** was activated by adding 5 equivalents of Et_6_Al_2_ at 273 K under an Ar atmosphere (see the “Optimized reaction conditions” section in Materials and Methods), and the formation of different intermediates was monitored in situ by EPR. On adding Et_6_Al_2_, the dark blue sample instantly changed color to orange brown. EPR spectra were taken at defined times after activation at 295 K (movie S1) and changed within minutes, showing a superposition of three different signals (Fig. 2). The corresponding powder spectra (i.e., frozen solutions) were recorded at 100 K for all three species. The spectra were broadened because of anisotropy in the *g* and *A* tensors, and therefore, the small hyperfine couplings were unresolved (fig. S1, left). Initial experiments at 10 K did not alter the spectra but slowed acquisition because of slower relaxation. Both frozen and liquid solution spectra (fig. S1) were simulated by a mixture of the three species using the spin Hamiltonian parameters given in table S1. Two of the room temperature spectra were in agreement with the previously reported species A (labeled A) with two magnetically equivalent ^31^P nuclei (nuclear spin, *I* = ½ and isotropic hyperfine coupling, ^P^*A*_iso_ = 52.3 MHz) resulting in a 1:2:1 triplet (indicated by dashed lines in green) and Cr^I^-bisarene with 10 near-equivalent protons (^1^H; *I* = ½; ^H^*A*_iso_ = 9.5 MHz) resulting in an undecet with 11 superhyperfine lines (with a binomial intensity distribution of 1:10:45:120:210:252:210:120:45:20:1), with the exception of the two outer lines being too weak to be observed. ^53^Cr (*I* = 3/2; natural abundance, 9.5%; ^Cr^*A*_iso_ = 50.5 MHz) causes quartet satellites where the undecet is further split into a 1:1:1:1 quartet. The inner two lines of the quartet overlap with the main multiplet and are not resolved. Cr^I^-bisarene displays the lowest *g* value (Fig. 2) (*26*). The signal at intermediate *g* value has not been reported and was labeled X (shown by dashed lines in blue). Although its *g* factor is similar to the one assigned to species D (Fig. 2) (*26*), here, the ^31^P coupling constant is smaller (two ^31^P nuclei; *I* = ½; ^P^*A*_iso_ = 76.0 MHz) and a further splitting into seven observable lines for each line in the triplet by an additional, even smaller, hyperfine coupling (*A*_iso_ = 8.2 MHz) is present (the presence of further outer lines cannot be excluded; Fig. 2, 30 min). This coupling arises from an even number of six or more magnetically equivalent spin ½ nuclei. It has been simulated using eight equivalent *I* = ½ nuclei. Species D would not be expected to exhibit this coupling pattern. Thus, the hypothesis of in situ formation of ethene complexes (Fig. 3) was tested by performing the identical experiment under ethene gas (1 bar).

**Fig. 4:**
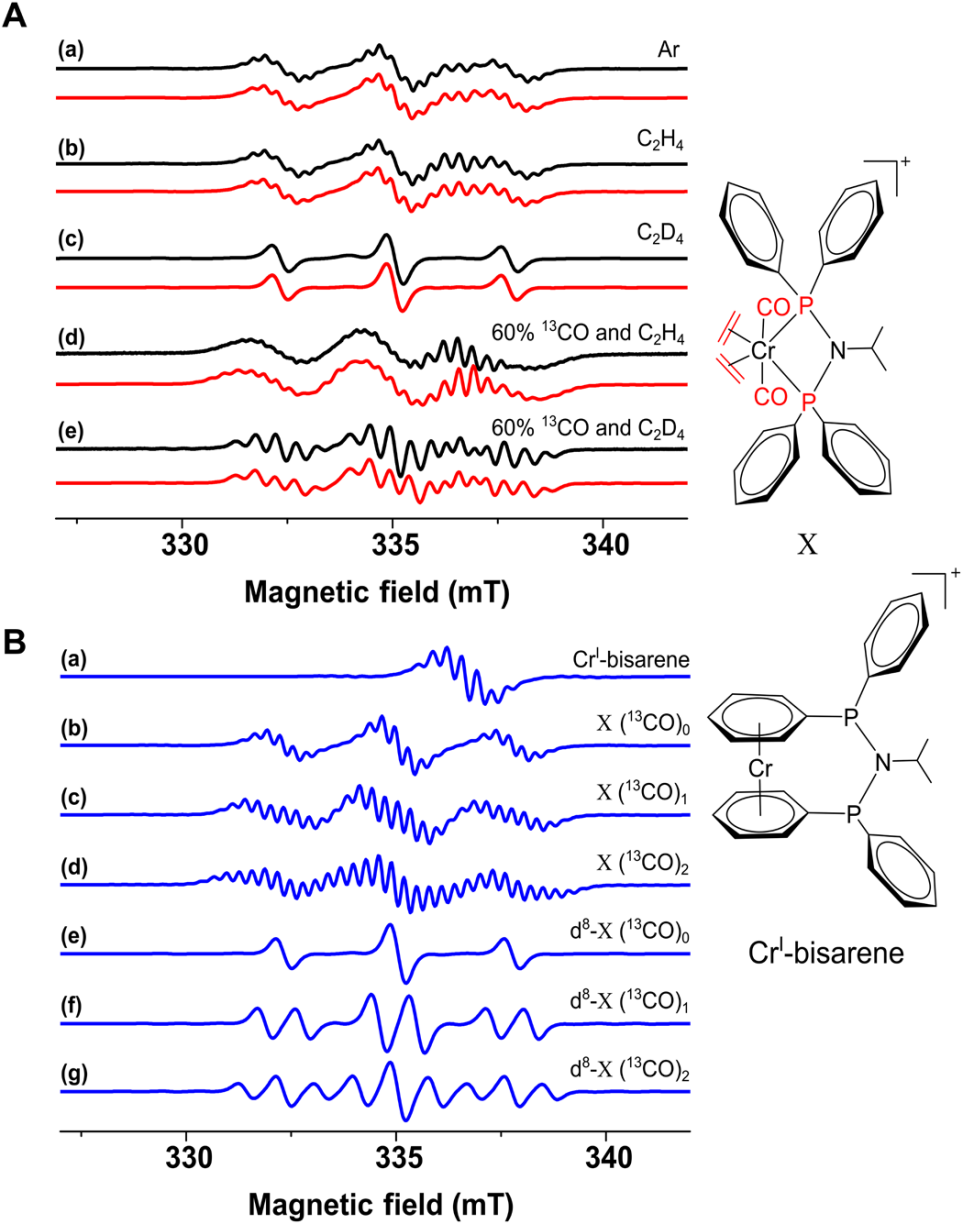
CW EPR data for X. (**A**) The CW EPR spectrum recorded for X at 295 K under identical conditions (a) Ar atmosphere, (b) ethene (1 bar), (c) deuterated ethene (1 bar), and subsequently, the activation of 60% ^13^CO enriched **1** was performed under (d) ethene (1 bar) and (e) deuterated ethene (1 bar). (**B**) The simulated EPR spectra for individual components are shown in blue (a) Cr^I^-bisarene (*g*_iso_ = 1.9875; 10 ^1^H nuclei: *I* = ½, ^H^*A*_iso_ = 9.5 MHz; one ^53^Cr nucleus: *I* = 3/2; natural abundance, 9.5%; ^Cr^*A*_iso_ = 50.5 MHz), (b) X (^13^CO)_0_ (two ^31^P and eight ^1^H nuclei), (c) X (^13^CO)_1_ (two ^31^P, eight ^1^H, and one ^13^C nuclei), (d) X (^13^CO)_2_ (two ^31^P, eight ^1^H, and two ^13^C nuclei), (e) d^8^-X (^13^CO)_0_ (two ^31^P and eight ^2^H nuclei), (f) d^8^-X (^13^CO)_1_ (two ^31^P, eight ^2^H, and one ^13^C nuclei), and (g) d^8^-X (^13^CO)_2_ (two ^31^P, eight ^2^H, and two ^13^C nuclei). Additional spin Hamiltonian parameters: *g*_iso_ = 1.9970; ^31^P nuclei: *I* = ½, ^P^*A*_iso_ = 76.0 MHz; ^1^H nuclei: *I* = ½, ^H^*A*_iso_ = 8.2 MHz; ^D^H nuclei: *I* = 1, ^D^*A*_iso_ = 1.3 MHz; and ^13^C nuclei: *I* = ½, ^C^*A*_iso_ = 25 MHz. The corresponding simulations are shown in red using the relative weightings for the individual components in blue (a) 0.25:0.75:0:0:0:0:0, (b) 0.3:0.7:0:0:0:0:0, (c) 0.02:0:0:0:0.98:0:0, (d) 0.3:0.112:0.336:0.252:0:0:0, and (e) 0.15:0:0:0:0.136:0.408:0.306, respectively.

An identical but more intense signal of X formed under ethene within 2 to 3 min [Fig. 4A (a and b) and fig. S2]. The experimental spectra can be simulated by a mixture of Cr^I^-bisarene and X (^13^CO)_0_ (Fig. 4B, a and b). However, activation under deuterated ethene (1 bar) saw the smaller hyperfine pattern collapse into the linewidth (Fig. 4A, c). This is reproduced by simulations (Fig. 4B, a and e) replacing hydrogen with deuterium (eight equivalent ^2^H nuclei, *I* = 1) nuclei and scaling the hyperfine coupling by the gyromagnetic ratios to 1.3 MHz (γ_H_/γ_D_ ≈ 6.5, γ_H_/γ_D_ ≈ 6.51, i.e., ^D^*A*_iso_ = ^H^*A*_iso_/6.5 = 1.3 MHz). This result indicates that X is an ethene bound Cr/PNP complex. One could speculate that the coupling to eight protons, which are replaced by deuterons upon introduction of d^4^-ethene, might originate from a d^8^-metallacyclopentane formed in situ. However, in this molecule, α and β protons would no longer be equivalent and would give rise to different hyperfine couplings. Furthermore, after oxidative cyclization, the chromium is in oxidation state III and would not be expected to have a doublet ground state. The resolution that we retrieve here has not been achieved with chromium(III) complexes in solution EPR where the additional zero-field splitting interaction broadens the lines substantially. Last, one could hypothesize a metallacyclopentane that has subsequently been reduced to an anionic chromium(I) complex. We have computed the expected proton hyperfine couplings (see the Supplementary Materials), which give much worse agreement with the experiment. On this basis, we rule out that species X is the metallacyclopentane and conclude that it has to be the bis-ethene complex. The hyperfine pattern resulting from eight equivalent ^1^H nuclei reproduces the experimental spectra and is consistent with two η^2^-ethene ligands (fig. S3). Gas chromatography–mass spectrometry (GC-MS) showed that the formation of 1-hexene and 1-octene is occurring under 1-bar ethene, demonstrating catalytic competence under the conditions stabilizing the bis-ethene intermediate (fig. S4). Moreover, GC-MS showed formation of ethene under in situ EPR conditions (fig. S5), explaining the formation of X in the absence of added ethene gas.

To inform on the fate of the CO ligands and identify the remainder of the chromium ligand sphere, ^13^C labeling of the carbonyl groups in **1** was performed and 60% ^13^C enrichment was confirmed by mass spectrometry and infrared spectroscopy (figs. S6 to S8). The activation under identical conditions as for natural abundance **1** resulted in additional lines for both C_2_H_4_ and C_2_D_4_, confirming the presence of CO as ligand (Fig. 4A, d and e). Spectral simulations (Fig. 4A) were performed as the weighted sum of different species and isotopologs expected for 60% ^13^CO enrichment (Fig. 4B). The isotopologs with 0, 1, and 2 ^13^CO ligands formed upon activation under deuterated ethene result in a singlet, doublet, and triplet splitting of each of the ^31^P triplet lines, respectively (Fig. 4B, e to g), producing a triplet of apparent quintets (Fig. 4A, e). The EPR spectrum upon activation under protonated ethene (Fig. 4A, d) was also found in agreement with the weighted sum of isotopologs with 0, 1, and 2 ^13^CO ligands (Fig 4B, b to d) and Cr^I^-bisarene. This confirms that two CO fragments remain bound while two are replaced by ethene ligands. The deconvolution of each spectral simulation is described in the Supplementary Materials (figs. S9 to S13), and the spin Hamiltonian parameters are listed in table S2. While alkylation of the carbonyls to form acyl ligands is possible in theory, these can be excluded because of a much larger density functional theory (DFT)–computed ^13^C hyperfine coupling not supported by the experiment (figs. S14 to S16 and tables S3 and S4).

## DISCUSSION

The combination of in situ EPR spectroscopy with selective isotope labeling of catalyst precursor and substrate allowed dissecting the composition of the transient species in the catalytically active mixture. All evidence agrees with X being the PNP bound cationic Cr^I^-bis(ethene)bis(CO) species ([Cr(C_2_H_4_)_2_(CO)_2_(PNP)]^+^). On the basis of the DFT studies, the structures of cis- and trans-CO isomers differed in energy by 84 kJ/mol, with the trans complex being more stable. In addition, this isomer shows better agreement between the DFT-calculated and experimental spin Hamiltonian parameters. In conclusion, the magnetic equivalence of both carbonyls and ethene protons hints at the symmetrical and most stable trans-CO isomer with fast rotation of ethene. This structural motif has been proposed early on as the crucial intermediate in the widely accepted catalytic mechanism. Nevertheless, for over four decades, no bis-ethene species of chromium has ever previously been detected. Here, this species was sufficiently stabilized to be observed at room temperature for tens of minutes, allowing EPR detection with high resolution. We attribute this to the two residual CO ligands enforcing a doublet ground state, as the low ratio of activator did not remove all CO ligands. The active catalyst most likely, as suggested in literature, would be without the carbonyl ligands, and the absence of experimental evidence of such a species is expected. The loss of carbonyl ligands on addition of excess Et_6_Al_2_ would lead to available vacant sites, resulting in higher activity and selectivity toward LAOs. In the future, activation of commercially used Cr^III^ precursors using a low amount of Et_6_Al_2_ in the presence of CO can be monitored to reproduce the bis-ethene intermediates. Investigation of bis-ethene intermediates might well develop to a platform for further improving catalyst design. Here, the low amounts of ethene either being generated in situ or added as nonpressurized gas resulted in “starved catalysis,” and this might well have contributed to the long lifetime of the bis-ethene species, allowing characterization by EPR. GC-MS showed that oligomerization is occurring under 1-bar ethene, demonstrating catalytic competence under the conditions stabilizing the bis-ethene intermediate.

In conclusion, the detection of a PNP bound cationic Cr^I^-bis(ethene) species is the first ever experimental detection of any bis-ethene complex of chromium. This crucial motif in the selective ethene oligomerization catalysis has been commonly accepted in the literature for over four decades but never directly identified. Its detection further points toward a Cr^I/III^ redox couple being active for ethene oligomerization. Thus, our results not only go beyond merely confirming the prior postulations but also move the science onto a much stronger and surer footing. Now that conditions for generating this species are known, it can be subjected to systematic investigation. The identification of conditions making it sufficiently persistent will aid the development of further improved catalytic systems for ethene oligomerization.

## MATERIALS AND METHODS

### Experimental design

#### Materials

Ag[Al(OC(CF_3_)_3_)_4_] was obtained from IoLiTec. PNP (*27*), Cr(CO)_4_(PNP), and **1** (*20*, *28*) were synthesized following the standard procedures as reported. The L-CAA (L-CAA = [(C_18_H_37_)_2_N(CH_3_)H][Al(OC(CF_3_)_3_)_4_]) salt was synthesized using the same procedure as for L-CAB (L-CAB = [(C_18_H_37_)_2_N(CH_3_)H][B(C_6_F_5_)_4_]) (*29*), except that (Et_2_O)_2.5_LiB(C_6_F_5_)_4_ was replaced by Li[Al(OC(CF_3_)_3_)_4_]. C_2_H_4_, C_2_D_4_ and ^13^CO were obtained from BOC. Et_6_Al_2_ was obtained from AkzoNobel, and all solvents were obtained from Sigma-Aldrich. Quartz EPR tubes (4 mm) from Wilmad, syringes from Hamilton, precision seal rubber septa from Sigma-Aldrich, and GC vials from Agilent Technologies were obtained.

#### Optimized reaction conditions

Our reaction design was optimized on the basis of several considerations: (i) The activator was directly injected into a vial containing the Cr^I^-precatalyst **1** for homogeneous mixing. (ii) The activation and the sample transfer to an EPR tube was performed at 273 K to slow down the reaction kinetics. (iii) Various solvent systems were tested, and a mixture of dichloromethane (DCM) and methylcyclohexane (MCH) (1:1 by volume) was selected. This mixture gave good resolution of the continuous-wave EPR signal at room temperature because addition of the alkane reduces the dielectric constant of the solvent mixture (solvents with large dielectric constant lead to microwave absorption and signal broadening). Furthermore, this solvent mixture forms a glass on freezing rather than crystallizing, thus giving higher sensitivity and resolution for EPR measurements when frozen. (iv) Activator (2.5 to 5 equivalents) is crucial for reproducibility of intermediates. (v) The reaction was performed under an argon atmosphere.

#### Sample preparation

All manipulations for chromium complexes were carried out in a glove box (MBraun LABmaster DP) under an argon atmosphere with oxygen and water below 0.1 parts per million using solvents purified and dried via standard procedures by passing through alumina and deoxygenated by freeze-pump-thaw cycles. A 13 mM stock solution of **1** (7.8 mg, 5 μmol) was prepared separately in a 2:1 mixture by volume of DCM and MCH referred to as Cr^I^-precatalyst solution. Aliquots (75 μl) of the Cr^I^-precatalyst solution were transferred into separate GC vials. A 0.4 M stock solution of Et_6_Al_2_ (0.11 ml, 0.4 mmol) was prepared separately in 2 ml of MCH. Aliquots (100 μl) of the Et_6_Al_2_ stock solution were transferred into separate GC vials. The Et_6_Al_2_ solution must be handled under an inert atmosphere, and for in situ activation, the concentration was kept below its pyrophoric limit (<0.7 M) for safe handling. The activation was performed in situ outside the glove box by injecting 25 μl of Et_6_Al_2_ solution (5 equivalents with respect to Cr^I^) into a GC vial containing Cr^I^-precatalyst solution at 273 K using an argon-flushed Hamilton syringe. The activated mixture was quickly transferred to a 4-mm quartz EPR tube (presealed with a rubber septum in the glove box) and immediately frozen at 77 K by transferring into liquid N_2_. A control sample for Cr^I^-precatalyst was also prepared in the glove box by replacing 25 μl of Et_6_Al_2_ stock solution with pure MCH, thereby keeping the same Cr^I^ concentration in all the samples. The activations were performed under identical conditions as above, except that a presealed tube with argon was flushed with 1 bar of ethene (C_2_H_4_ or C_2_D_4_) using a gas/vacuum manifold before the activation.

^13^C isotope enrichment of the carbonyl groups was performed by heating a Cr(CO)_4_(PNP) solution to 150°C in ^13^CO (2 bars). This complex was oxidized to the ^13^C-enriched **1** with Ag[Al(OC(CF_3_)_3_)_4_] following standard procedures, and activation was performed under identical conditions as above for the natural abundance **1**.

#### EPR spectroscopy

The EPR spectra were acquired using an X-band (~9.34 GHz) Bruker EMX 10/12 spectrometer equipped with an ELEXSYS super-high-sensitivity probehead (Bruker ER4122SHQE) and an ESR900 He cryostat. The sample temperature was stabilized to 100 K with a variable temperature helium flow cryostat (Oxford Instruments) using liquid N_2_. The spectra were obtained using 10-mW microwave power, 0.1-mT modulation amplitude, 40-ms sampling time, and 100-kHz modulation frequency. The solution state spectra were obtained at 295 K. For monitoring the spectral changes over time, incremental scans were performed at 295 K with a delay of 60 s. All simulations were made using EasySpin 5.0.7 (*30*) with the functions “pepper” for frozen sate and “garlic” for isotropic spectra measured in solution state.

## SUPPLEMENTARY MATERIALS

Supplementary material for this article is available at http://advances.sciencemag.org/cgi/content/full/6/51/eabd7057/DC1
